# Bier’s spots with onset in childhood*

**DOI:** 10.1590/abd1806-4841.20164533

**Published:** 2016

**Authors:** Larissa Karine Leite Portocarrero, Maria Isabel Ramos Saraiva, Marcella Amaral Horta Barbosa, Isis Suga Veronez, Bethania Cabral Cavalli Swiczar, Neusa Yuriko Sakai Valente

**Affiliations:** 1 Universidade de São Paulo (USP) – São Paulo (SP), Brazil; 2 Private clinic – Juiz de Fora (MG), Brazil; 3 Private clinic – São Paulo (SP), Brazil; 4 Hospital do Servidor Público Estadual (HSPE) – São Paulo (SP), Brazil

**Keywords:** Blood Vessels, Muscle, smooth, vascular, Skin diseases

## Abstract

Bier spots are small, irregular, hypopigmented macules that are usually found on the arms and legs. The macules disappear when the limb is raised. Bier spots have been reported in association with a number of conditions but there is no consistent association to specific desease. Although they usually affect young adults, we report a case of Bier spots that began in childhood. As an asymptomatic and possibly transitional condition, the disease does not require treatment.

## INTRODUCTION

Bier spots are asymptomatic, small, irregular, hypochromic macules surrounded by an erythematous-cyanotic area found most frequently on the limbs. Bier spots represent a distinct pattern of vascular mottling that affect mostly young adults, ranging from 20- 40 years of age. There is no consensus on the pathophysiogenesis of Bier spots.^1^ A striking feature is the disappearance of the lesions with the rising of the limbs or diascopy, and accentuation of the macules when limbs are hanging down. We report a case of Bier spots with onset in childhood.

## CASE REPORT

**Figure 1:**
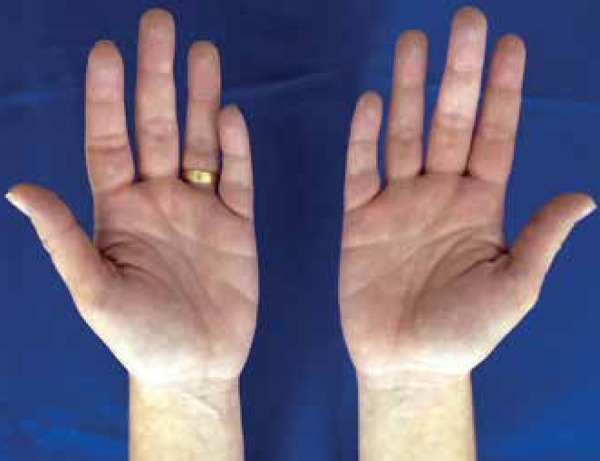
Hypochromic spots on the hands that appear on limbs in pendent position

**Figure 2:**
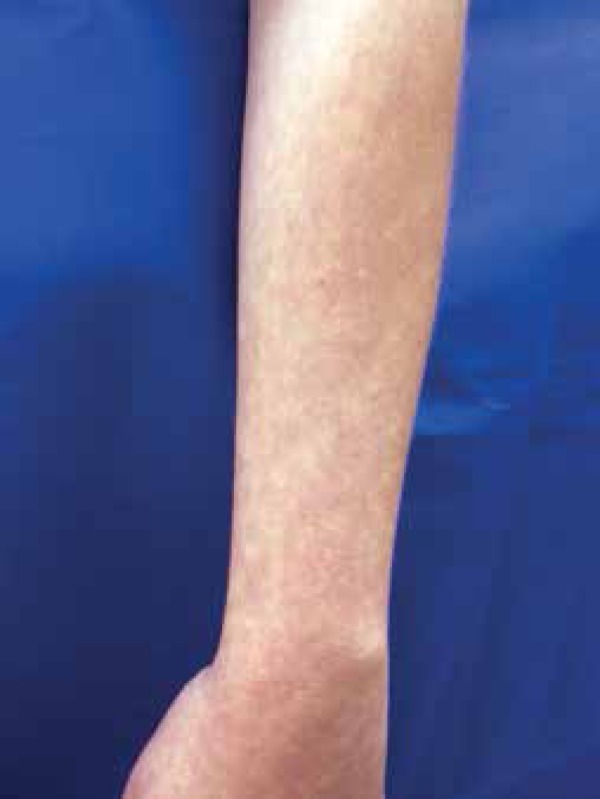
Similar hypochromic spots are observed on the pendent forearm

We report a 29-year-old female patient who presented with asymptomatic hypopigmented macules on her upper limbs since she was four. She reported that the macules become more visible when the arms hang down and disappear when the arms are raised (Figures 1 and 2). Complementary exams revealed iron deficiency anemia, positive ANA-Hep2 (1/160) with a fine speckled pattern, and low levels of protein C (57.1) (reference value: 70-140). Beta 2 microglobulin, anti-ENA, anti-native DNA, syphilis serology, cryoglobulins, anticardiolipin, PCR, and metabolic panel were all normal.

## DISCUSSION

Bier spots were first described in 1898.^2^ Through an experiment, Bier noted that the spots appeared under external occlusion of venous blood flow to the forearm and disappeared when the flow was restored.^3^ Although the disease commonly occurs in adulthood, with only few cases reported in adolescents so far, our patient presented the onset at age 4.^4^

There is no consensus on the pathogenesis of Bier spots. Some authors suggest that the disease is a vascular anomaly caused by vasoconstriction of small vessels. Hence, there is venodilation in erythematous areas and venoconstriction in pale areas. The spots are regarded as an exaggerated physiologic vasoconstrictive response and are induced by venous stasis-associated hypoxia or, conversely, by a failure of the venoarteriolar reflex in dermal ascending arterioles in response to venous filling.^5,6^ Khera and English III suggested that the term “physiological anemic macules” better reflects this mechanism.^7^

Clinical diagnosis is made by observing the disappearance of the hypochromic macules upon limb elevation. In some reported cases, the spots become more visible after emotional stress and less visible after physical activity.^1^ Since histopathology shows no abnormality, skin biopsy is unnecessary.

The prevalence of this condition is unknown. Considering its asymptomatic clinical picture, the disease can be underestimated.^8^

Differential diagnosis includes other hypochromic diseases, such as pityriasis versicolor, vitiligo, nevus despigmentosus, postinflammatory hypopigmentation, and anemic nevus.

Although there is no well-established association in the literature with specific diseases, some cases were related to palmar hyperhidrosis, insomnia, pregnancy, cryoglobulinemia, scleroderma renal crisis, aortic arch hypoplasia, lichen planus, alopecia areata, and Peutz Jeghers syndrome.^9^ Our patient showed positive ANA and protein C below the reference range, but with no other symptoms or related clinical signs. These results were interpreted as isolated laboratory abnormalities.

The disease is asymptomatic and requires no treatment. Although the macules appear transiently depending on the position of the limbs, they tend to be chronic.^7^

Although Bier spots are usually idiopathic and benign, diagnosis is important because, in addition to reports of associated systemic diseases, they can mimic other conditions amenable to treatment.
